# Do Critically Ill Patients Undergoing Continuous Renal Replacement Therapy Require Ceftaroline Dosage Adjustments? Ceftaroline PopPK Model and Dosage Simulations with the Probability of Target Attainment Analysis Based on Retrospective Data †

**DOI:** 10.3390/antibiotics14040347

**Published:** 2025-03-27

**Authors:** Arkadiusz Adamiszak, Krzysztof Pietrzkiewicz, Alicja Bartkowska-Śniatkowska, Piotr Smuszkiewicz, Krzysztof Kusza, Edmund Grześkowiak, Agnieszka Bienert

**Affiliations:** 1 Department of Pharmacology, Poznan University of Medical Sciences, 60-806 Poznan, Poland; agbienert@ump.edu.pl; 2 Doctoral School, Poznan University of Medical Sciences, 60-812 Poznan, Poland; 3 Department of Paediatric Anaesthesiology and Intensive Therapy, Poznan University of Medical Sciences, 60-572 Poznan, Poland; kpietrzkiewicz@ump.edu.pl (K.P.); asniatko@ump.edu.pl (A.B.-Ś.); 4 Department of Anesthesiology, Intensive Therapy and Pain Management, Poznan University of Medical Sciences, 60-352 Poznan, Poland; piotr.smuszkiewicz@icloud.com (P.S.); k-kusza@ump.edu.pl (K.K.); 5 Department of Clinical Pharmacy and Biopharmacy, Poznan University of Medical Sciences, 60-806 Poznan, Poland; grzesko@ump.edu.pl

**Keywords:** ceftaroline, continuous renal replacement therapy, population pharmacokinetics, Monte Carlo simulations, probability of target attainment, intensive care unit

## Abstract

**Objectives**: We aimed to develop a population pharmacokinetic (PopPK) model and evaluate dosing regimens for different renal clearances and continuous renal replacement therapy (CRRT) settings. **Methods**: Data were collected from four studies in intensive care unit (ICU) adult patients receiving 400–600 mg of ceftaroline every 8–12 h in a one-hour infusion. The PopPK model was developed according to non-linear mixed effects modeling implemented in Monolix 2024R1. To investigate dosing recommendations, Monte Carlo simulations and probability of target attainment (PTA) analysis were performed in Simulx 2024R1. **Results**: We collected 296 plasma concentrations from 29 non-CRRT patients and 24 pre-filter (systemic), 23 post-filter, and 23 effluent concentrations from four CRRT patients using WebPlotDigitizer (Version 4.7). A five-compartment model, with the first-order elimination from the central compartment and additional elimination with the effluent during CRRT, best described the ceftaroline concentrations. Creatinine clearance (*Cl_Cr_*) was identified as a covariate on the clearance of elimination (*Cl*) and CRRT modality on the central and peripheral compartments’ volumes and intercompartmental clearance. The results of dosage simulations for different CRRT modalities and *Cl_Cr_*, *S. pneumoniae* (MIC = 0.25 mg/L) and methicillin-resistant *S. aureus* (MRSA) (MIC = 1 mg/L) infections, and assumed 100%ƒT_>MIC_ target, revealed that registered ceftaroline dosages are sufficient to achieve assumed PTA, except MRSA infection in patients with augmented renal clearance (ARC). **Conclusions**: Our successfully developed model allows flexible PK simulations of ceftaroline, including real-time changes in settings and even temporary or permanent cessation of CRRT. However, the results of our study warrant clinical validation and should be used with caution primarily due to the limited CRRT patient number included in the analysis.

## 1. Introduction

Ceftaroline fosamil, a ceftaroline prodrug, is a fifth-generation cephalosporin reserved for treating resistant gram-positive and gram-negative bacteria, including methicillin-resistant *Staphylococcus aureus* (MRSA) and penicillin-resistant *Streptococcus pneumoniae* [1,2]. Immediately after the start of the infusion, ceftaroline fosamil is almost completely converted to ceftaroline, which is approximately 20% bound to plasma proteins, and about 65% is eliminated renally [3,4,5]. The standard pharmacokinetic/pharmacodynamic (PK/PD) target for β-lactams assumes that the concentration of antibiotic for the whole time exceeds the minimum inhibitory concentration (MIC) of the target bacteria (100%ƒT_>MIC_). In turn, for severe conditions, especially related to critically ill patients, a four times more rigorous target equal to 100%ƒT_>4xMIC_ is proposed [3,6].

In the case of intensive care unit (ICU) patients, many clinical implications may influence antibiotics pharmacokinetic (PK), leading to dosage corrections [7,8]. Augmented renal clearance (ARC), defined as creatinine clearance (*Cl_Cr_*) >130 mL/min/1.73 m^2^, related to sepsis, shock, and critical condition, leads to increased clearance of renally eliminated medications and, as a consequence, to sub-therapeutic concentrations [9,10]. Moreover, sepsis and/or septic shock often lead to acute kidney injury (AKI), which in most critically ill patients requires initiation of continuous renal replacement therapy (CRRT) [4,6]. The combined effect of intensive care patients’ conditions and CRRT affecting antibiotic elimination makes PK complex and challenging to predict, complicating dose adjustments [6,11].

To our knowledge, no dosing standards have been recommended by The European Committee on Antimicrobial Susceptibility Testing (EUCAST) or Stanford Health Care Antimicrobial Dosing Reference Guide for ceftaroline in patients undergoing CRRT [12,13]. This study aimed to develop a population pharmacokinetic (PopPK) model and investigate dosages for ICU patients on CRRT with the assessment of whether registered ceftaroline dosages are sufficient to achieve assumed PTA for *S. pneumoniae* and MRSA.

## 2. Results

### 2.1. Patient Characteristics

A total of 296 plasma concentrations from 29 adult ICU patients, and 24 pre-filter (systemic), 23 post-filter, and 23 effluent concentrations from four adult ICU patients undergoing CRRT were considered in the analysis. Eight concentrations below the limit of quantification were calculated according to the M3 censoring method implemented into Monolix 2024R1 [14]. The characteristics of the studied population are presented in Table 1.

### 2.2. Pharmacokinetic Model Building

**Figure 1:**
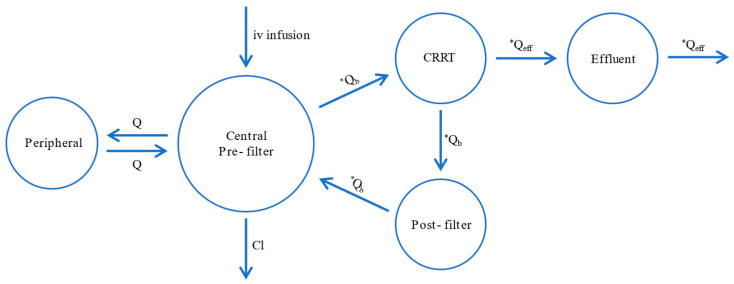
Ceftaroline five-compartment model according to Al-Shaer et al. approach [15,16]. * When a patient is not under CRRT, all flow rates are set to zero. CRRT—continuous renal replacement therapy; *Cl*—elimination clearance; *Q*—inter-compartmental clearance; *Q_b_*—blood flow rate; *Q_eff_*—total effluent flow rate.

**Figure 2:**
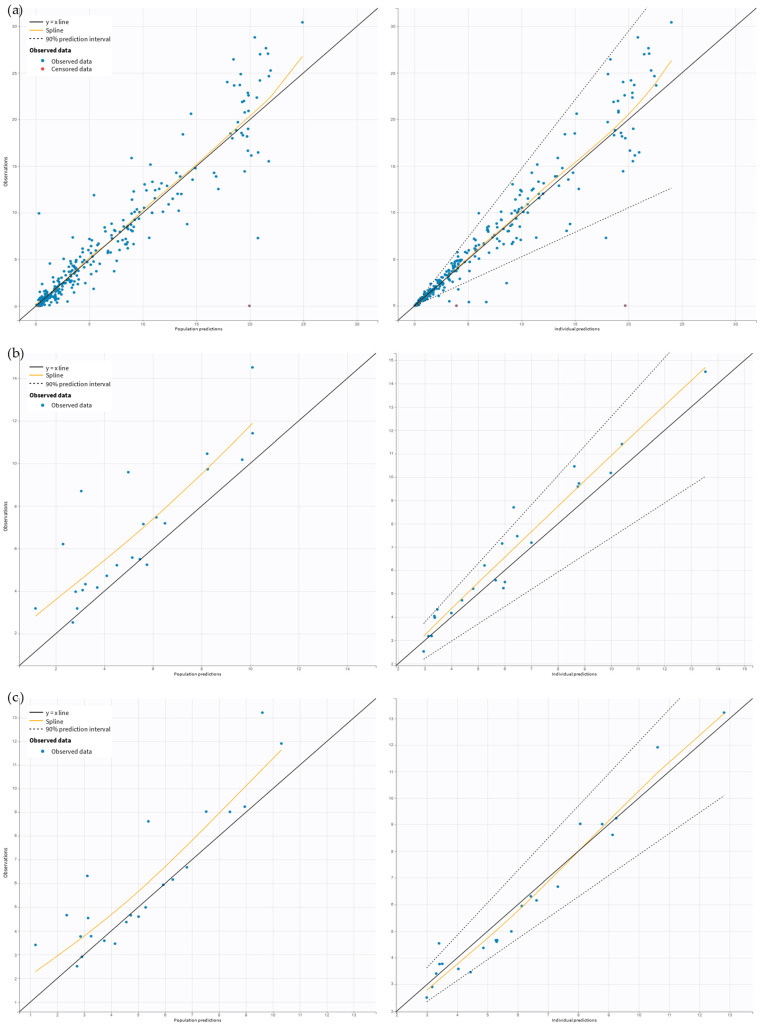
Observations versus predictions plots. The following panels are devoted to (**a**) pre-filter (systemic) concentrations, (**b**) post-filter concentrations, and (**c**) effluent concentrations. The figures on the left in each panel present the relationship between observed drug concentrations (*Y*-axis) versus population predicted concentrations (*X*-axis). The figures on the right in each panel present the relationship between observed drug concentrations (*Y*-axis) versus individual predicted concentrations (*X*-axis).

**Figure 3:**
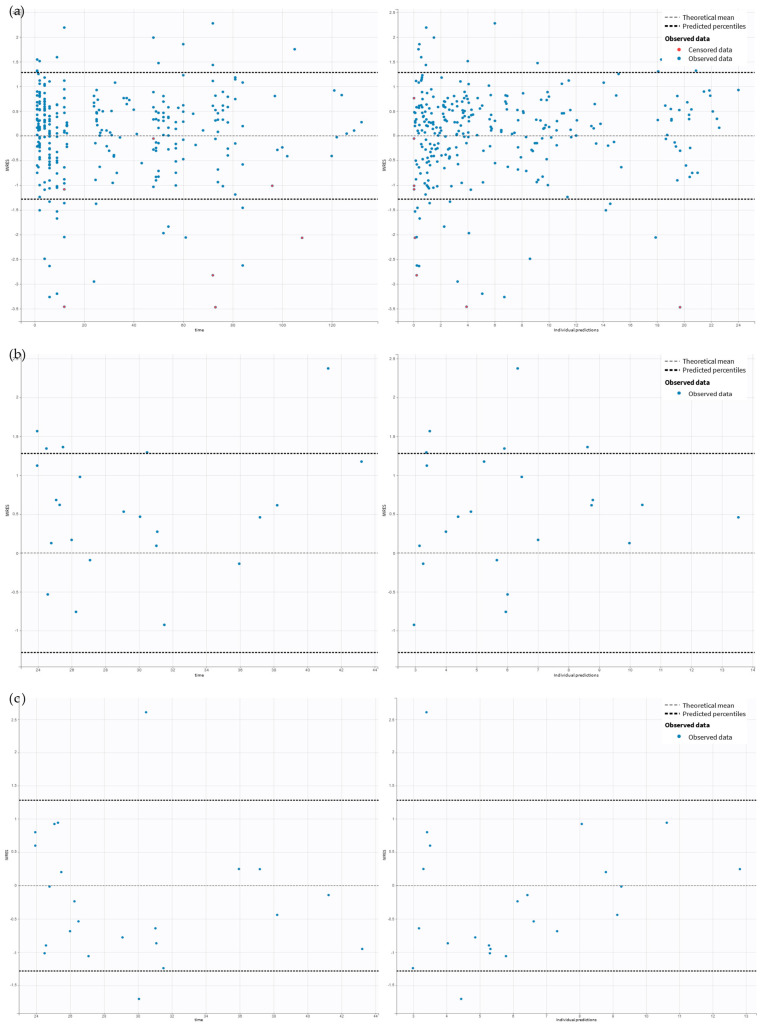
Residuals’ scatter plots. The following panels are devoted to (**a**) pre-filter (systemic) concentrations, (**b**) post-filter concentrations, and (**c**) effluent concentrations. The figures on the left in each panel present the individual weighted residuals (IWRES) (*Y*-axis) versus time (*X*-axis). The figures on the right in each panel present the individual weighted residuals (IWRES) (*Y*-axis) versus individual predicted concentrations (*X*-axis).

**Figure 4:**
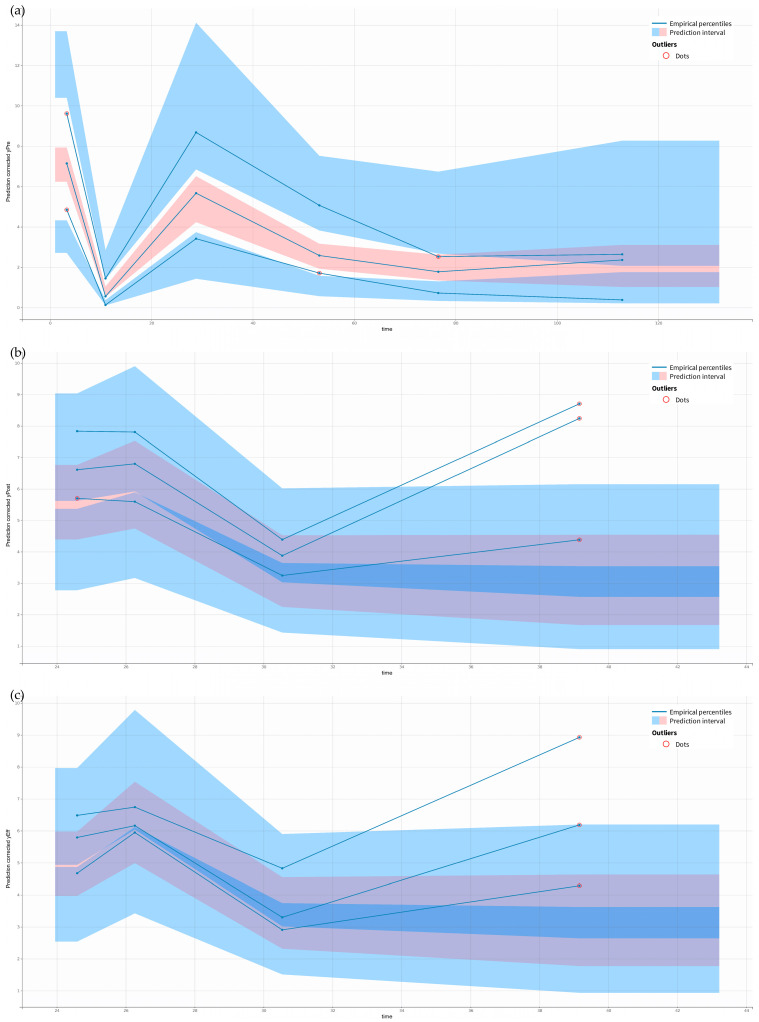
pcVPC plots. The following panels are devoted to (**a**) pre-filter (systemic) concentrations, (**b**) post-filter concentrations, and (**c**) effluent concentrations.

Ceftaroline pre-filter (systemic), post-filter, and effluent concentrations were well described by the proposed five-compartment model with the first-order elimination from the central compartment and, in the case of patients undergoing CRRT, additional elimination with the effluent (Figure 1). Due to the instability of the model, the individual variability (*IIV*) was estimated for the volume of distribution of the central (*V_Central_*) and peripheral (*V_Peripheral_*) compartments, and inter-compartmental clearance (*Q*) and inter-occasion variability (*IOV*) was estimated for the elimination clearance from the central compartment (*Cl*). The proportional error models for pre-filter (systemic), post-filter, and effluent concentrations resulted in the best data fit. In the final model, four covariates significantly improved the model estimations. At each step of adding covariates, the objective function value (OFV) dropped more than 6.63 (*p* < 0.01). The *Cl* increased by 57% in patients with high *Cl_Cr_* (309 mL/min/1.73 m^2^) compared to the median *Cl_Cr_* (159 mL/min/1.73 m^2^). Continuous venovenous hemodialysis (CVVHD) increased *V_Central_* by 60%, while continuous venovenous hemodiafiltration (CVVHDF) decreased *V_Central_* by 52%. Patients undergoing CRRT had 269% higher *V_Peripheral_* and *Q* compared to patients without CRRT. The inclusion of the mentioned covariates resulted in a decrease in the *IOV* of *Cl_Cr_* by 13% and in the *IIV* of *V_Central_* and *Q* by 21% and 34%, respectively. The final model estimates are presented in Table 2. The prediction-corrected visual predictive check (pcVPC) and goodness-of-fit (GOF) for the final model indicated a good description of the data and no major model misspecification. The points in the observed vs. predicted plots are symmetrically clustered around the line, indicating no evident trends (Figure 2). The residuals are distributed around zero, and most points are within the range of −2 and 2 (Figure 3). The pcVPC for pre-filter (systemic), post-filter, and effluent ceftaroline concentrations showed that most observed concentrations were within the predicted intervals (Figure 4). The mlxtran code of the final model, along with graphs of the distribution of the residuals, the distribution of the individual parameters, the distribution of the standardized random effects, the correlation between random effects, the individual parameters vs. covariates, and stochastic approximation expectation-maximization (SAEM), are presented in the Supplementary Materials.

### 2.3. Monte Carlo Simulations

We successfully investigated PTA for assumed dosing schemes and MICs from 0.0625 to 4 mg/L. The ready-to-use Simulx 2024R1 files with simulations based on the final model and the results of all simulated scenarios are presented in the Supplementary Materials. The results of simulations for indications posted in the summary of product characteristics (SmPC) related to MRSA and *Streptococcus pneumoniae*-caused infections and MICs considered as breakpoints in the EUCAST breakpoint and QC tables [12] are shown in Table 3.

## 3. Discussion

**Figure 5:**
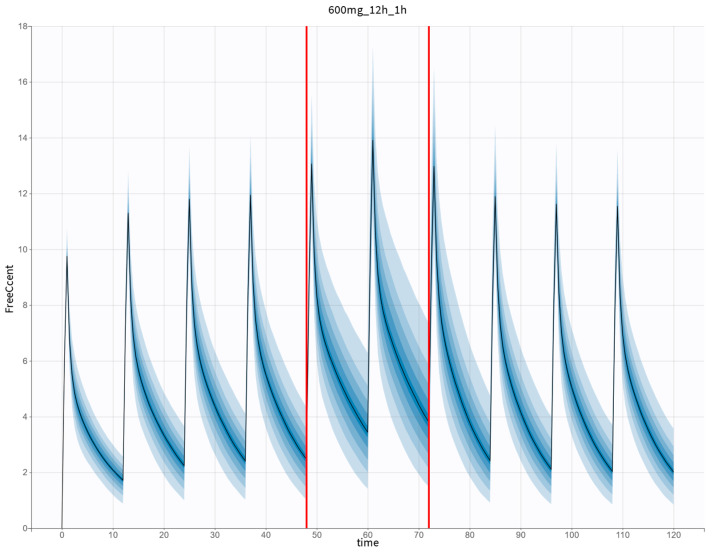
Concentration vs. time profile for the standard dosage of 600 mg per 12 h in 1 h infusion simulated for theoretical patients undergoing CVVHD with *Q_eff_* equal to 2.5 L/h stopped for 24 h at 48 h of ceftaroline administration and then continued with *Q_eff_* of 4.0 L/h. Vertical solid lines, the continuous vertical lines representing the time when CRRT was switched off. CVVHD—continuous venovenous hemodialysis; *Q_eff_*—total effluent flow rate.

Our study successfully used a slightly modified five-compartment model developed for cefepime by Al-Shaer et al. [15,16] to analyze the PopPK of ceftaroline administered to ICU patients undergoing or not CRRT. To our knowledge, this is the first PopPK model of ceftaroline developed considering CRRT modality and machine flow rates, which also describes its PK in patients without CRRT with different renal functions. The model is characterized by a high level of flexibility, allowing real-time changes in the blood flow rate (*Q_b_*) and the total effluent flow rate (*Q_eff_*) and the situation of CRRT cessation or no CRRT scenario to be taken into account (Figure 5).

Ceftaroline population *Cl* from *V_central_* for a non-CRRT median ICU patient (Table 1) was 11.2 L/h. Our result is consistent with the *Cl* values reported for the same patient populations in Chauzy et al.’s PopPK studies [17,18], which were 11.3 and 10.6 L/h, respectively. In turn, the mean estimated total clearance for patients undergoing CRRT was 5.66 L/h + assumed *Q_eff_*, which coincides with the average result (6.68 L/h) reported by the Kalaria et al. team [4]. Similar observations concern *Q* values of 8.80, 6.06, and 6.79 L/h in our and Chauzy et al. models, respectively [17,18]. Differences in *V_central_* and *V_peripheral_* values in our model vs. Chauzy et al. models [17,18] might stem from different model structures caused by the addition of CRRT compartments and simplification of the model by omitting the metabolism step of prodrug ceftaroline fosamil to ceftaroline, not including its concentrations in the cerebrospinal fluid and the PK of the ceftaroline-M1 metabolite.

The proposed dosages of ceftaroline for 100%ƒT_>MIC_ target and investigated *Cl_Cr_* values are consistent with those demonstrated in Chauzy et al.’s study [18]. We did not perform continuous infusion simulations due to the limited 6 h in room temperature ceftaroline solution stability indicated in SmPC [5]. As an extension of Chauzy et al.’s [18] simulation results, we performed additional simulations, including each variant of dosages mentioned in SmPC and a more rigoristic PK/PD target of 100%ƒT_>4xMIC_. To compare dosages for CRRT patients with those proposed by Kalaria et al. [4], we performed additional simulations for the same 50%ƒT_>MIC_ PK/PD target, assuming MIC = 1 mg/L. The results of the mentioned simulations are reported in the Supplementary Materials (Table S1). The dosages simulated for CVVHD patients and Qeff between 2.5 and 4.0 L/h are consistent with that proposed by Kalaria et al. [4]. However, in the case of CVVHDF, we suggest a higher dosage (600 mg per 12 h in 1 h infusion) for *Q_eff_* = 4.0 L/h.

The results presented in Table 3 and delivered with the article and Supplementary Materials (especially PTA charts and Simulx 2024R1 file) allow the choice or simulation of dosages for particular patients’ cases; however, they should be used cautiously due to the lack of clinical validation of the model.

The study’s main limitation is the limited number of patients undergoing CRRT. With this in mind, direct generalization of our study’s results against patients undergoing CRRT is biased and should be interpreted cautiously. While a small number of patients allows for the establishment of some general characteristics, it may not be sufficient to represent the entire CRRT patient population. Another limitation is the measurement of the total fraction of ceftaroline with the omission of free fraction determinations and a restricted number of tested covariates. A broader database of potential covariates and determinations of the free ceftaroline fraction could allow estimation of the *IIV* of the *Cl*. In reference to data digitizing with WebPlotDigitizer [19], studies to date confirm the relatively high precision of the extracted data, indicating an error of 0.22% to 8.92% [20,21]. The accuracy and precision mostly depend on the quality of the graph and program calibration before extraction [19,20,21]. In conclusion, the data extraction method did not pose a high risk of error at the model development and estimation stage, provided that the data were reliably represented on the graphs and the program used for data extraction was correctly calibrated. Finally, it is worth noting that the proposed doses are based on Monte Carlo simulations using the final model estimations and need to be evaluated in a clinical setting.

## 4. Materials and Methods

### 4.1. Study Population

Pharmacokinetic and clinical data were retrospectively obtained from four articles describing the treatment of ICU patients with 400–600 mg of ceftaroline every 8–12 h in a one-hour infusion [4,17,18,22]. The PK data from the graphs were extracted using WebPlotDigitizer (Version 4.7) [19].

### 4.2. Pharmacokinetic Analysis

Ceftaroline concentration-time data were analyzed using PopPK modeling according to the SAEM and Markov Chain Monte Carlo algorithms for non-linear mixed effects models implemented in the Monolix 2024R1 [14]. Given the incorporation of data from two population approach models, a two-compartment patient model with linear elimination was assumed a priori, in alignment with the findings of the studies as mentioned before [17,18,23]. In turn, patients undergoing CRRT with post-filter and ultrafiltrate ceftaroline concentrations were included in the study by the slightly modified methodology proposed by Al-Shaer et al. [15,16], which involved the addition of three compartments related to the CRRT machine (Figure 1).

The transfer of ceftaroline through the CRRT compartments was described as the following formula [15]:

$$k\;[h^{-1}]\;=\;\frac{Flow\;rate\;[L/h]}{V\;[L]}$$

where k is the rate of transfer, *V* is the volume of a given compartment, and the flow rate is *Q_b_* or *Q_eff_*. The PK parameters inter-individual variability (*IIV*) and inter-occasion variability (*IOV*) were assumed to be log-normally distributed. The proportional and combined error models for describing the residual unexplained variability were evaluated.

The covariates analyzed were determined by their availability in the articles from which the PK data were extracted [4,17,18,22]. In order to exclude the influence of data from different studies, we additionally added a categorical variable identifying data from a specific study. The age and weight were tested as a baseline, continuous covariates, and gender and CRRT modality were treated as categorical. In turn, for 18 patients from the Chauzy et al. study [18], two *Cl_Cr_* measurements were achievable per patient, so we treated *Cl_Cr_* values as a continuous covariate with *IOV*. Covariates were added to a model according to the Conditional Sampling use for the Stepwise Approach based on the Correlation tests (COSSAC) approach implemented in Monolix 2024R1 [14,24]. The effect of covariates was evaluated using the following equations.

For continuous covariates:

$$\theta_{i}=\theta_{pop}\times {\left(\frac{{COV}_{i}}{{median}_{{COV}_{i}}}\right)}^{\theta_{cov}}\times e^{\eta_{i}}\;$$

For categorical covariates:

$$\theta_{i}=\theta_{pop}\times e^{\theta_{cov}}\times e^{\eta_{i}}$$

where *θ_i_* represents the individual parameter estimate, *θ_pop_* is the population estimated value for this parameter, *COV_i_* corresponds to the individual value of a covariate, *θ_cov_* is the estimated effect of that covariate on the parameter, and *η_i_* is equal to the individual value of the random effect associated to the parameter describing the difference between the population value of the parameter and the individual value of that parameter for *i*th subject.

Model selection was based on a decrease of at least 3.84 points (*p* < 0.05) for 1 degree of freedom in corrected Bayesian Information Criteria (BICc), OFV defined as −2 × Log Likelihood of the data, the stability of the model, the precision of the parameter estimates, and GOF diagnostic plots evaluated at each step of the building process. Additionally, in the case of the covariate model, the decision to test a given covariate in the model was based on assessing the relationships between random effects and covariates using Pearson’s correlation test for continuous data and ANOVA for categorical data [25]. In turn, the assessment of the necessity of dropping a covariate from the model was undertaken based on the correlation test, which evaluates whether the coefficient of influence of a given covariate on a parameter is significantly different from zero, and the results of the Wald test [25]. The final model was validated by the pcVPC.

### 4.3. Simulations

The concept of Monte Carlo simulations involves generating a large number (thousands) of virtual patients by randomly sampling from the probability distribution of the PopPK model estimates. The aforementioned approach allows for the testing of different therapeutic approaches, such as evaluating alternative dosing scenarios and predicting probable treatment outcomes, such as achieving therapeutic targets [26,27].

In our study, simulations of dosing regimens based on the final model were performed in Simulx 2024R1 [28]. We tested 200/300/400/600 mg in a 1 h infusion every 12 h and a 2 h infusion every 8 h as standard and high dosages according to the SmPC, respectively [5]. The dosage of 800 mg was tested according to Chauzy et al.’s suggestion [18]. For each combination, we simulated 2500 virtual patients as 50 patients in a group replicated 50 times, accounting for the same individuals among simulated groups. During the PTA analysis, we assumed 100%*ƒ*T_>MIC_ and 100%ƒT_>4xMIC_ as standard and high β-lactam pharmacokinetic/pharmacodynamic (PK/PD) targets, respectively [3,6]. The PTA ≥ 90% for MIC values up to 4 mg/L was considered an acceptable probability of success [12,29,30,31]. The free fraction of ceftaroline was calculated based on a percentage (~20%) of protein binding mentioned in SmPC [5]. In line with the results of Chauzy et al.’s study [18], we investigated scenarios for patients without CRRT with *Cl_Cr_* equal to 80, 130, 210, and 300 mL/min/1.73 m^2^. In turn, for patients undergoing CRRT, we assumed a median *Cl_Cr_* of 58 mL/min/1.73 m^2^ and median *Q_b_* of 15 L/h for the simulation analysis and tested *Q_eff_* of 2.5, 3.0, 3.5, and 4.0 L/h.

## 5. Conclusions

Both *Cl_Cr_* and *Q_eff_* influence the elimination of ceftaroline, leading to the need for changes in the dosages to achieve assumed PK/PD targets for different MIC values of the bacteria. Patients undergoing CRRT need lower than standard (600 mg q12 h Tinf 1 h) ceftaroline dosages. In turn, for patients with ARC and *Cl_Cr_* ≥ 210 mL/min/1.73 m^2^, no registered/proposed dosages are enabled to reach the assumed PTA (except 100%ƒT > MIC target for *Streptococcus pneumoniae* and MIC lower than 0.25 mg/L). Our study supplements information on the variability of ceftaroline’s PK due to varying renal elimination in different clinical situations involving ICU patients. Future perspectives regarding the development and application of our model include the addition of more patients, especially those undergoing CRRT, and external clinical validation.

## Figures and Tables

**Table 1:** Patients’ characteristics (n = 33).

Characteristics	Median [Range] or Number (%)
Gender	
Female	12 (36%)
Male	21 (64%)
Age (years)	51 [21–77]
Weight (kg)	75.5 [49–111.8]
*Cl_Cr_* (mL/min/1.73 m^2^)	159 [42–309]
CRRT	
Yes	4 (12%)
No	29 (88%)
CRRT modality	
CVVHD	2 (50%)
CVVHDF	2 (50%)
*Q_b_* (mL/min)	250 [250–300]
*Q_eff_* (mL/h)	3200 [2700–3660]

*Cl_Cr_*—creatinine clearance estimated by the Modification of Diet in Renal Disease (MDRD) formula; CRRT—continuous renal replacement therapy; CVVHD—continuous venovenous hemodialysis; CVVHDF—continuous venovenous hemodiafiltration; *Q_b_*—blood flow rate; *Q_eff_*—total effluent flow rate.

**Table 2:** Estimates of the base and final models for ceftaroline.

Parameters	Mean Estimate (RSE%)	
	Base Model	Final Model
*Cl* (L/h)	10.27 (7.0)	11.23 (5.7)
*Cl_Cr_* on *Cl*	–	0.68 (16.1)
*IOV* on *Cl* (%CV)	48.12 (10.6)	34.96 (12.3)
*V_Central_* (L)	21.26 (13.2)	20.04 (4.1)
CVVHD on *V_Central_*	–	0.47 (18.2)
CVVHDF on *V_Central_*	–	−0.42 (21.3)
*IIV* on *V_Central_* (%CV)	24.44 (44.3)	3.70 (57.8)
*Q* (L/h)	8.51 (25.9)	8.80 (20.7)
CRRT on *Q*	–	0.99 (26.6)
*IIV* on *Q* (%CV)	50.69 (38.3)	16.40 (58.5)
*V_Peripheral_* (L)	16.72 (11.2)	16.85 (10.5)
CRRT on *V_Peripheral_*	–	0.99 (30.5)
*IIV* on *V_Peripheral_* (%CV)	38.92 (29.0)	38.60 (20.8)
*V_CRRT_* (L)	2.18 (14.9)	2.69 (42.3)
*V_Effluent_* (L)	0.44 (17.4)	0.60 (6.1)
*V_Post-filter_* (L)	1.29 (4.23)	1.15 (3.5)
*bPre*	0.29 (5.0)	0.29 (5.0)
*bPost*	0.18 (20.6)	0.16 (21.0)
*bEff*	0.17 (17.3)	0.13 (16.9)

*bPre*—proportional error of pre-filter (systemic) concentration; *bPost*—proportional error of post-filter concentration; *bEff*—proportional error of effluent concentration; *Cl*—elimination clearance; *Cl_Cr_*—creatinine clearance; CRRT—continuous renal replacement therapy; CVVHD—continuous venovenous hemodialysis; CVVHDF—continuous venovenous hemodiafiltration; *IIV*—inter-individual variability; *IOV*—inter-occasion variability; *Q*—inter-compartmental clearance; *V_Central_*—the volume of distribution of the central compartment; *V_Peripheral_*—the volume of distribution of the peripheral compartment n; *V_CRRT_*—the volume of distribution of CRRT theoretical compartment; *V_Effluent_*—the volume of distribution of effluent theoretical compartment; *V_Post-filter_*—the volume of distribution of post-filter theoretical compartment.

**Table 3:** Ceftaroline dosing recommendations according to CRRT modality and *Q_eff_*, renal function, and PK/PD target for *Streptococcus pneumonia* and Methicillin-resistant *Staphylococcus aureus* EUCAST breakpoints, based on the lowest daily doses required to achieve at least 90% target attainment.

	*Streptococcus pneumoniae* (MIC = 0.25 mg/L)		Methicillin-Resistant *Staphylococcus aureus* (MIC = 1 mg/L)	
	100%ƒT_>MIC_	100%ƒT_>4xMIC_	100%ƒT_>MIC_	100%ƒT_>4xMIC_
With CRRT				
CVVHD				
*Q_eff_* = 2.5 L/h	200 mg q12 h T_inf_ 1 h	200 mg q8 h T_inf_ 2 h	200 mg q8 h T_inf_ 2 h	800 mg q8 h T_inf_ 2 h
*Q_eff_* = 3.0 L/h	200 mg q12 h T_inf_ 1 h	300 mg q8 h T_inf_ 2 h	300 mg q8 h T_inf_ 2 h	–
*Q_eff_* = 3.5 L/h	200 mg q12 h T_inf_ 1 h	300 mg q8 h T_inf_ 2 h	300 mg q8 h T_inf_ 2 h	–
*Q_eff_* = 4.0 L/h	200 mg q12 h T_inf_ 1 h	300 mg q8 h T_inf_ 2 h	300 mg q8 h T_inf_ 2 h	–
CVVHDF				
*Q_eff_* = 2.5 L/h	200 mg q12 h T_inf_ 1 h	300 mg q8 h T_inf_ 2 h	300 mg q8 h T_inf_ 2 h	–
*Q_eff_* = 3.0 L/h	200 mg q12 h T_inf_ 1 h	300 mg q8 h T_inf_ 2 h	300 mg q8 h T_inf_ 2 h	–
*Q_eff_* = 3.5 L/h	200 mg q12 h T_inf_ 1 h	300 mg q8 h T_inf_ 2 h	300 mg q8 h T_inf_ 2 h	–
*Q_eff_* = 4.0 L/h	300 mg q12 h T_inf_ 1 h	400 mg q8 h T_inf_ 2 h	400 mg q8 h T_inf_ 2 h	–
Without CRRT				
*Cl_Cr_*				
80 mL/min/1.73 m^2^	300 mg q12 h T_inf_ 1 h	400 mg q8 h T_inf_ 2 h	400 mg q8 h T_inf_ 2 h	–
130 mL/min/1.73 m^2^	200 mg q8 h T_inf_ 2 h	800 mg q8 h T_inf_ 2 h	800 mg q8 h T_inf_ 2 h	–
210 mL/min/1.73 m^2^	600 mg q8 h T_inf_ 2 h	–	–	–
300 mL/min/1.73 m^2^	800 mg q8 h T_inf_ 2 h	–	–	–

“–”—target attainment has not been achieved with tested dosing schemes; *Cl_Cr_*—creatinine clearance; CRRT—continuous renal replacement therapy; CVVHD—continuous venovenous hemodialysis; CVVHDF—continuous venovenous hemodiafiltration; MIC—minimum inhibitory concentration; T_inf_—time of infusion; *Q_eff_*—total effluent flow rate.

## Data Availability

The data presented in this study are available in the Supplementary Materials.

## Supplementary Materials

The following supporting information can be downloaded at: https://www.mdpi.com/article/10.3390/antibiotics14040347/s1, Figure S1: Distribution of residuals. (a) Pre-filter concentrations. (b) Post-filter concentrations. (c) Effluent concentrations; Figure S2: Model parameters distribution; Figure S3: Random effects plots; Figure S4: Correlations of random effects; Figure S5: Covariates diagnostics plots; Figure S6: Stochastic approximation expectation-maximization (SAEM) plots; Figure S7: Creatinine clearance effect on a typical individual PK; Figure S8: The CVVHD/CVVHDF effect on a typical individual PK; Table S1: Ceftaroline dosage simulations for CRRT patients assuming 50%ƒT > MIC and 100% PTA and MIC = 1 mg/L; Final Model Syntax; External hyperlink to the GitHub repository with the ready-to-use Simulx2024R1 file based on the final model and PTA charts.

## Abbreviations

The following abbreviations are used in this manuscript:

AKI	Acute kidney injury
ARC	Augmented renal clearance
BICc	corrected Bayesian Information Criteria
CRRT	Continuous renal replacement therapy
*Cl*	Climination clearance
*Cl_Cr_*	Creatinine clearance
COSSAC	COnditional Sampling use for the Stepwise Approach
CVVHD	Continuous venovenous hemodialysis
CVVHDF	Continuous venovenous hemodiafiltration
EUCAST	The European Committee on Antimicrobial Susceptibility Testing
GOF	Goodness of fit
ICU	Intensive care unit
*IIV*	Inter-individual variability
*IOV*	Inter-occasion variability
MIC	Minimum inhibitory concentration
MRSA	Methicillin-resistant *Staphylococcus aureus*
OFV	Objective function value
pcVPC	Prediction-corrected visual predictive check
PK	Pharmacokinetic
PK/PD	pharmacokinetic/pharmacodynamic
PopPK	Population pharmacokinetic
PTA	Probability of target attainment
SAEM	Stochastic approximation expectation-maximization
SmPC	Summary of product characteristics
*Q*	Inter-compartmental clearance
*Q_b_*	Blood flow rate
*Q_eff_*	Effluent flow rate
*V_Central_*	Volume of the central compartment
*V_Peripheral_*	Volume of the peripheral compartment
