# Supplementary material for: Do Critically Ill Patients Undergoing Continuous Renal Replacement Therapy Require Ceftaroline Dosage Adjustments? Ceftaroline PopPK Model and Dosage Simulations with the Probability of Target Attainment Analysis Based on Retrospective Data
Source: Antibiotics (Basel). 2025 Mar 27;14(4):347. doi: 10.3390/antibiotics14040347 (PMC12024021; doi:10.3390/antibiotics14040347)
Supplement: Supplementary file 1 [file antibiotics-14-00347-s001.zip › antibiotics-3508795-supplementary.pdf]

Supplementary materials for "Do Critically Ill Patients Undergoing Continuous Renal Replacement Therapy Require Ceftaroline Dosage Adjustments? Ceftaroline PopPK Model and Dosage Simulations with the Probability of Target Attainment Analysis Based on Retrospective Data."

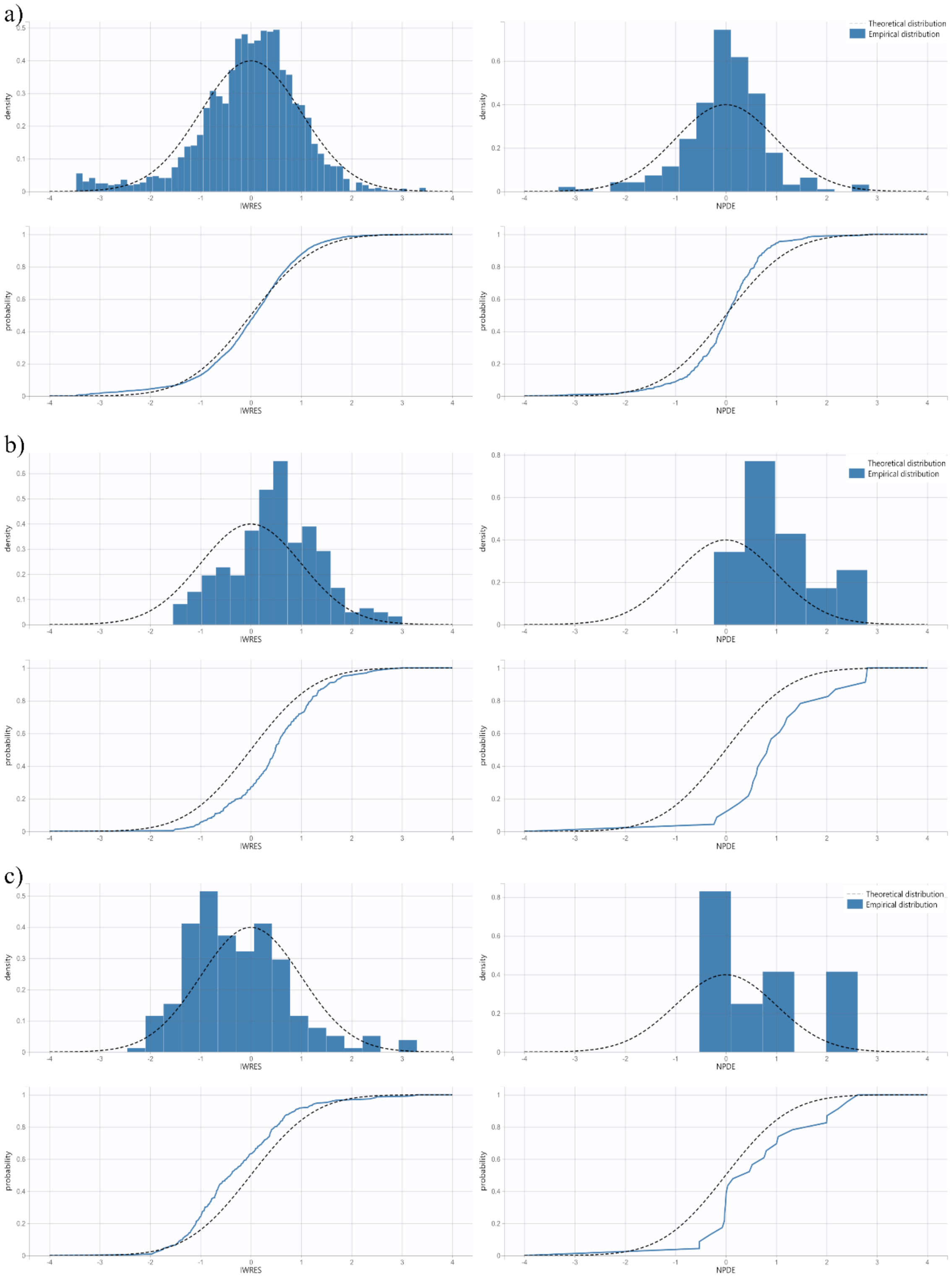

Figure S1. Distribution of residuals. a) Pre-filter concentrations. b) Post-filter concentrations. c) Effluent concentrations.

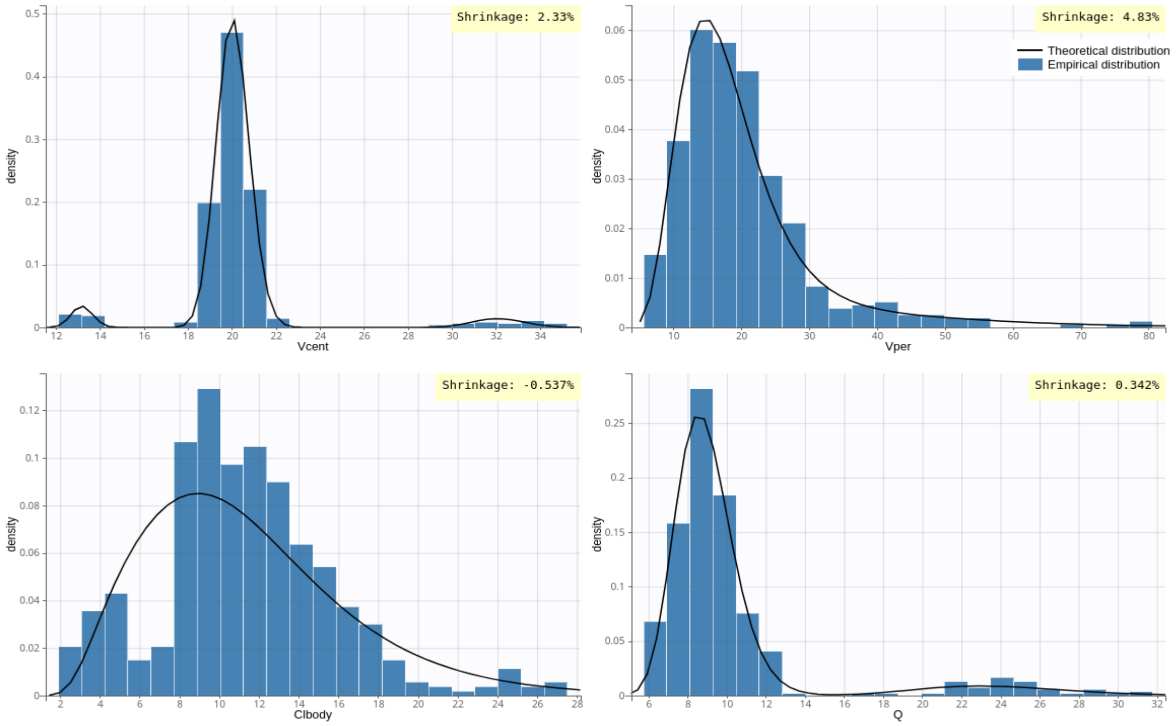

Figure S2. Model parameters distribution.

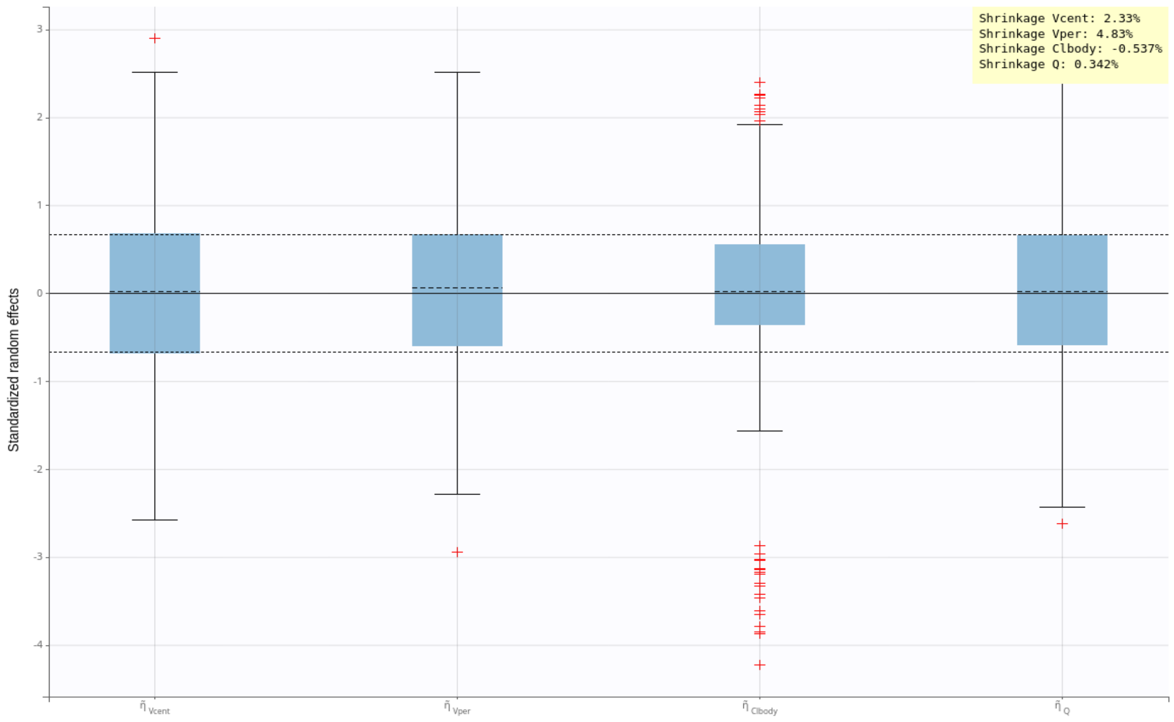

Figure S3. Random effects plots.

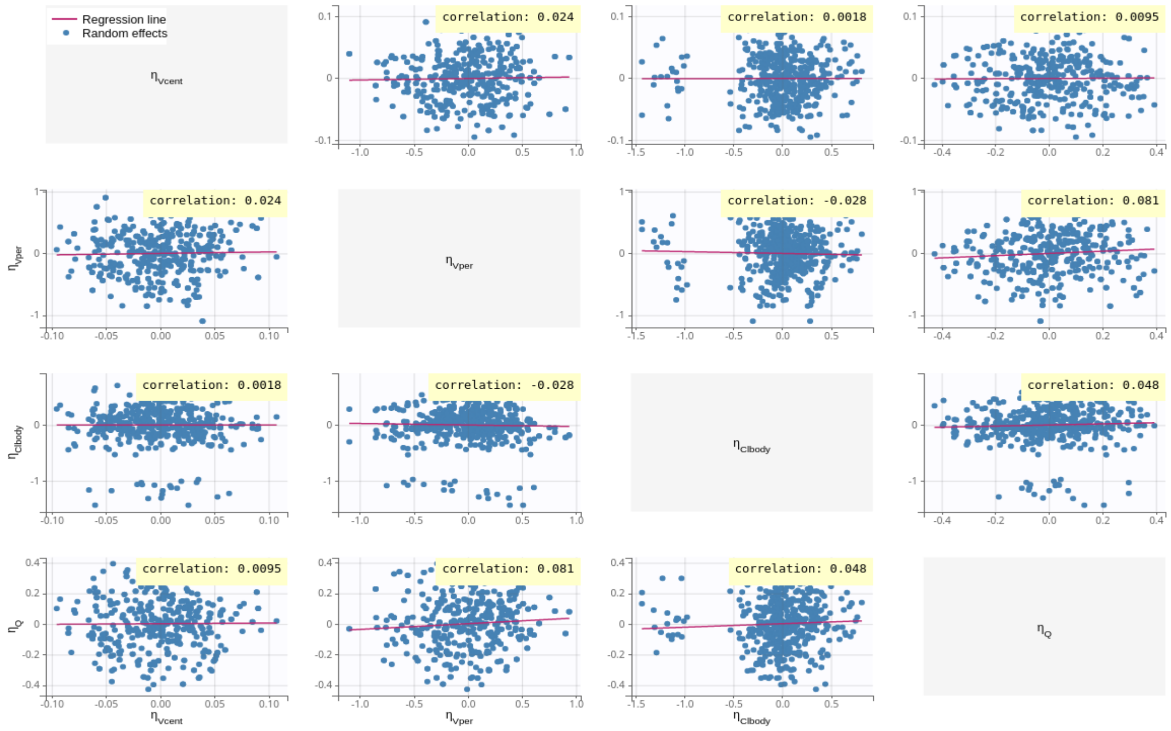

Figure S4. Correlations of random effects.

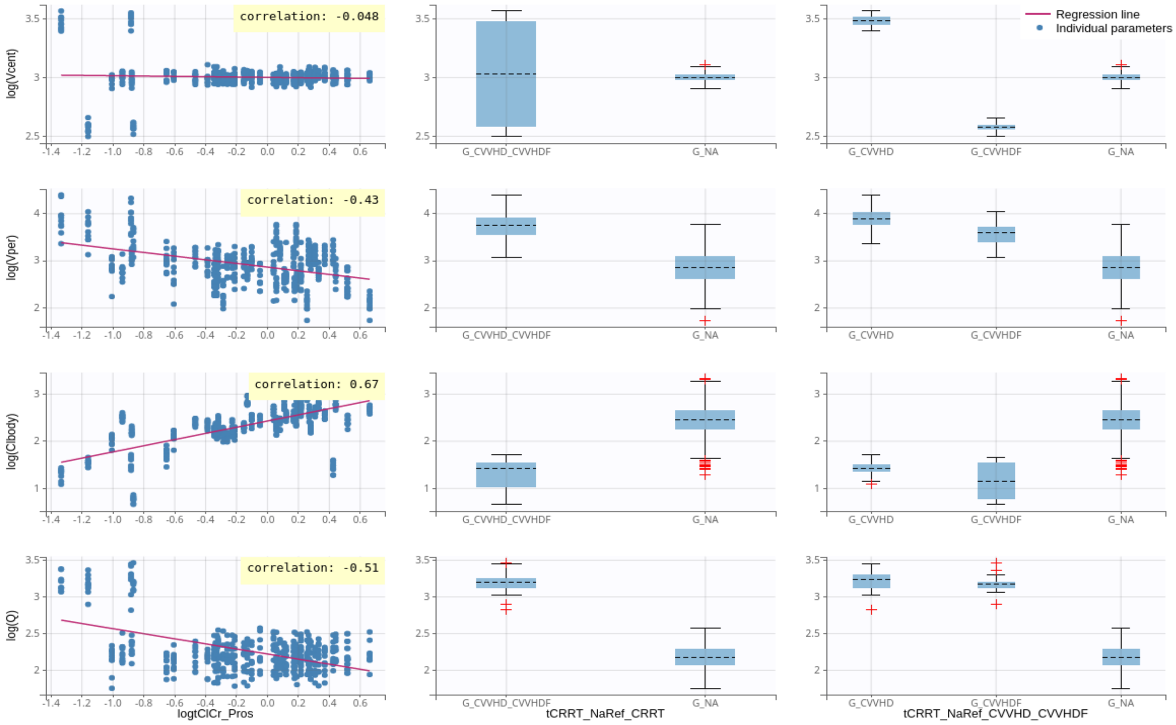

Figure S5. Covariates diagnostics plots.

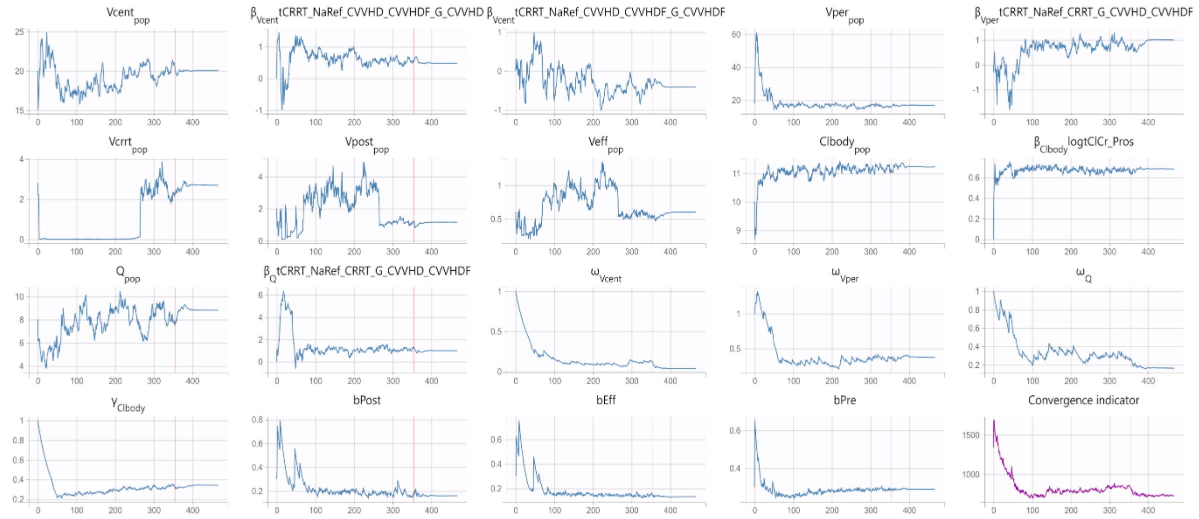

Figure S6. Stochastic approximation expectation-maximization (SAEM) plots.

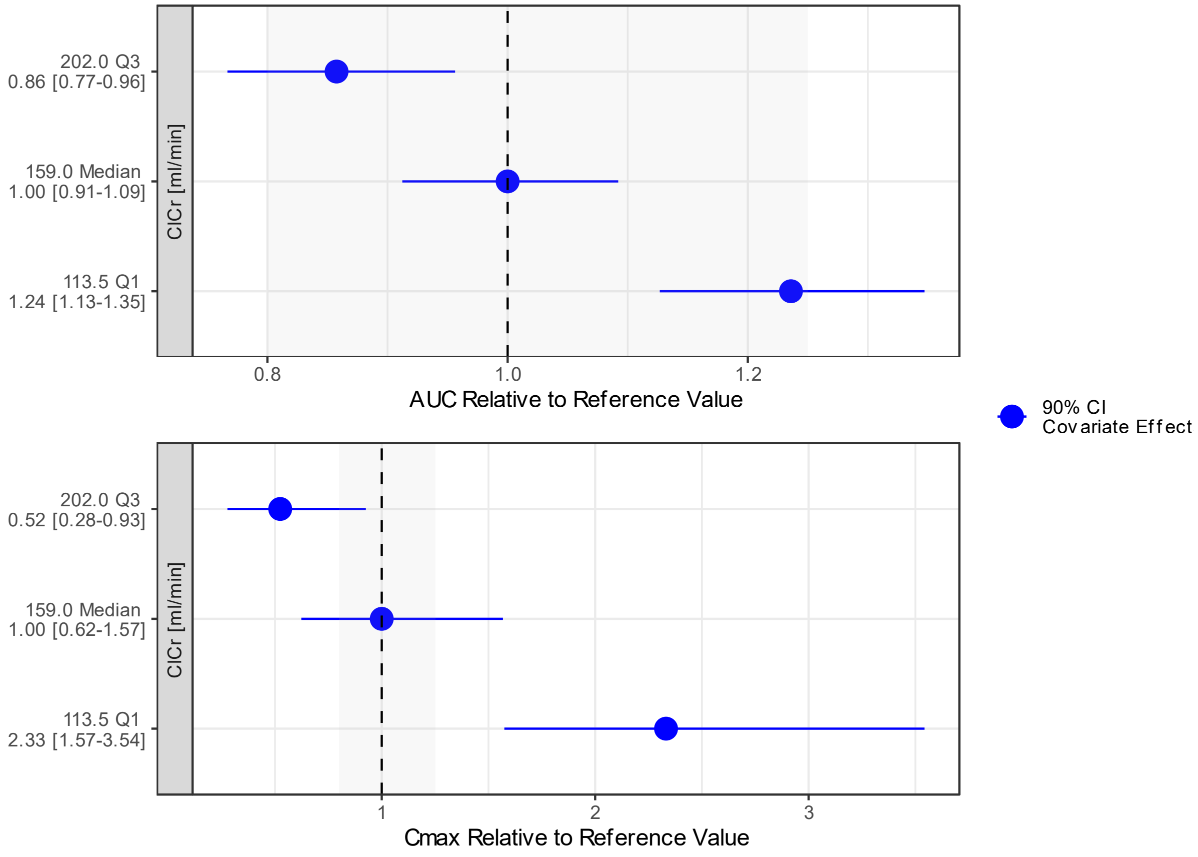

Figure S7. The effect of creatinine clearance (Cl_{Cr}) on AUC (Top) and C_{max} (Bottom) of typical individuals simulated based on the final model.

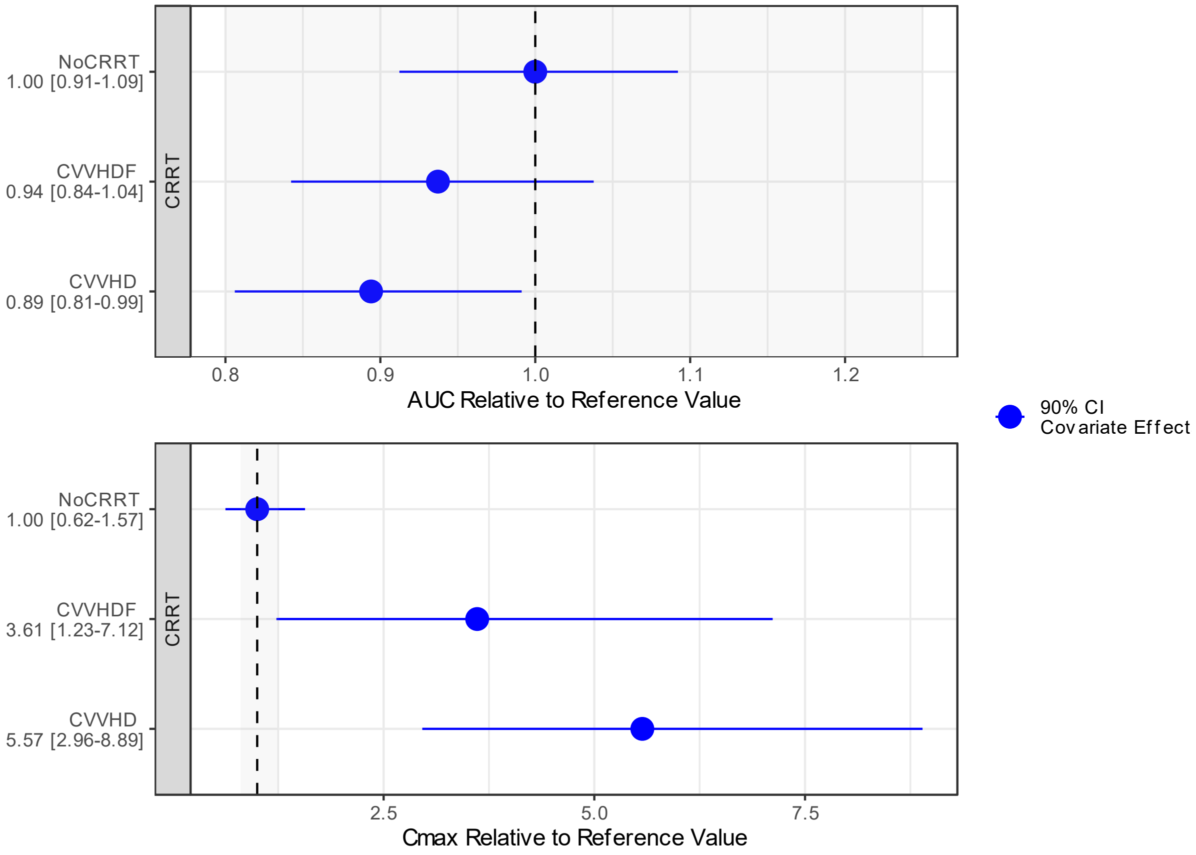

Figure S8. The effect of continuous renal replacement therapy modality (CVVHD or CVVHDF) on AUC (Top) and C_{max} (Bottom) of typical individuals simulated based on the final model.

Table S1. Ceftaroline dosage simulations for CRRT patients assuming 50%$fT_{>MIC}$ and 100% PTA and MIC = 1 mg/L.

Q_{eff}	CVVHD	CVVHDF
2.5 L/h	400mg q12h T_{inf} 1h	400mg q12h T_{inf} 1h
3.0 L/h	400mg q12h T_{inf} 1h	400mg q12h T_{inf} 1h
3.5 L/h	400mg q12h T_{inf} 1h	400mg q12h T_{inf} 1h
4.0 L/h	400mg q12h T_{inf} 1h	600mg q12h T_{inf} 1h

CRRT, continuous renal replacement therapy; CVVHD, continuous venovenous hemodialysis; CVVHDF, continuous venovenous hemodiafiltration; MIC, minimum inhibitory concentration; PTA, probability of target attainment; $T_{inf,}$ time of infusion; Q_{eff}, total effluent flow rate.

Monolix model syntax

```
[LONGITUDINAL]
input = {Vcent, Vper, Vcrrt, Vpost, Veff, Clbody, Q,  IS_CRRT, Qeff, Qb}
IS_CRRT = {use=regressor}
Qb = {use=regressor}
Qeff = {use=regressor}

PK:
depot(target = Acent)

EQUATION:
kelbody = Clbody/Vcent
k12 = Q/Vcent
k21 = Q/Vper
k13 = Qb/Vcent
k41 = Qb/Vpost
k35 = Qeff/Vcrrt
k34 = Qb/Vcrrt
kelcrrt = Qeff/Veff
Ccent = Acent/Vcent
Cpost = Apost/Vpost
Ceff = Aeff/Veff

ddt_Acent = -kelbody*Acent - k12*Acent + k21*Aper + (IS_CRRT * (- k13*Acent + k41*Apost))
ddt_Aper = k12*Acent - k21*Aper
ddt_Acrrt = IS_CRRT * (k13*Acent - k35*Acrrt - k34*Acrrt)
ddt_Apost = IS_CRRT * (k34*Acrrt - k41*Apost)
ddt_Aeff = IS_CRRT * (k35*Acrrt - kelcrrt*Aeff)

OUTPUT:
output = {Ccent, Cpost, Ceff}
```

External hyperlink

The ready-to-use Simulx2024R1 file based on the final model and PTA charts are reachable at the GitHub repository (link below).

https://github.com/arkadiusz-adamiszak/Ceftaroline_ICU_Retrospective.git
